# Analysis of age-specific vulnerability to mental disorders among U.S.-bound Chinese immigration applicants in Eastern China: A retrospective study using routinely collected health data

**DOI:** 10.1097/MD.0000000000047223

**Published:** 2026-01-23

**Authors:** Zhiying Ju, Xuan Zhou, Peiqin Zhu, Peng Li, Weixin Wang, Jia Qin

**Affiliations:** a Department of Radiology, Shanghai International Travel Healthcare Center (Shanghai Customs Port Clinic), Shanghai, China.

**Keywords:** China, cross-sectional study, immigrants, mental disorder

## Abstract

Although mental health challenges in immigrant populations are well-recognized, data on mental disorders among United States (U.S.) immigration applicants remain scarce. To address this scarcity, we retrospectively analyzed health assessment data (2017–2024) from Chinese–American immigration applicants in Eastern China. We addressed a critical gap by providing the first large-scale data on the prevalence and characteristics of these applicants. Data were obtained from health assessments conducted at the Shanghai International Travel Healthcare Center between 2017 and 2024. Applicants diagnosed with mental disorders were included. Analyzed variables encompassed gender, age, marital status, temporal trends in detection rates, and disorder distribution. Of 34,558 applicants (mean age 32.7 ± 11.2 years), 54 were diagnosed with mental disorders (prevalence: 1.56‰). No significant gender differences were observed (χ^2^ = 0.53, *P* = .463). Marital status distribution indicated that 66.67% (n = 36) were married, and 33.33% (n = 18) were unmarried. Age-specific analysis revealed elevated rates in applicants aged ≤15, 15–30, and ≥75 years (χ^2^ = 19.352, *P* = .002). Neurodevelopmental disorders were most common (39%), followed by schizophrenia spectrum disorders (15%), depressive disorders (13%), and neurocognitive disorders (11%). Bipolar disorders (7%), anxiety disorders (4%), and other conditions (7%) were less frequent. Temporal trends from 2017 to 2024 showed no significant change (Cochran–Armitage *Z* = −0.4004, *P* = .6889), with an increase from 2017 to 2021, followed by a decrease from 2021 to 2024. Findings reveal a distinct vulnerability in younger (≤30 years) and older (≥75 years) applicants and identify neurodevelopmental disorders as the most prevalent condition. These novel insights underscore the urgent need for and provide a foundation for implementing targeted, age-stratified mental health screening and early intervention protocols within the immigration health assessment process. This is crucial for improving the well-being of this understudied population and ensuring equitable access to care.

## 1. Introduction

Mental disorders represent a significant global health burden, with lifetime prevalence rates estimated between 15% and 36%,^[1]^ which is gradually gaining traction among immigrant populations. Over 280 million international migrants worldwide face compounded stressors spanning pre-departure trauma, acculturative strain, and systemic exclusion by 2025. Migration is becoming increasingly recognized as an important influencing factor in psychological vulnerability. This global context underscores specific risks for subpopulations, as evidenced by the National Latino and Asian American Studies study documenting a 17.3% lifetime prevalence of psychiatric disorders among Asian Americans. Notably, significant heterogeneity exists within this group: for instance, Chinese immigrants exhibit elevated acculturative stress linked to English proficiency, migration age, and racial discrimination – factors buffered not by conventional support systems but by social status attainment.^[2]^

The Chinese–American immigrant cohort exemplifies these systemic challenges. As the largest Asian-origin group in the United States (U.S.) (growing from <1% to 15% of the population since 1990), their mental health profiles reveal unique patterns.^[3]^ Specifically, the mental health of immigrant populations is not merely an individual concern, but a public health imperative that directly impacts national healthcare expenditures, social integration outcomes, and health equity goals. Consequently, the rising number of migrants poses substantial challenges for healthcare systems worldwide. Given that the health status of all social groups, including migrants, directly affects national healthcare expenditures and resource utilization, many countries emphasize addressing their health needs. This recognition explains why scholarly and clinical attention has increasingly turned to the accessibility of mental health services, particularly for immigrant communities.^[4–7]^

This study’s timeliness is reflected in the rapid growth of China’s immigrant population, changes in immigration policies, and the urgency of the impact of global events such as the Coronavirus Disease 2019 epidemic.^[8,9]^ Additionally, migrant students are at risk of mental health problems and social isolation caused by the Coronavirus Disease 2019 epidemic. As a result of the epidemic, there was a loss of a sense of belonging and long-term psychological trauma, as well as higher levels of anxiety and depression. There was also a sense of loneliness during the isolation period, leading to difficulties in social reconstruction when classes resumed. These converging factors may exacerbate psychological stress among applicants, making research particularly urgent. Despite heightened interest in immigrant health, the mental health status of Chinese–American immigration applicants manifests as a gap: pre-migration mental health data are virtually absent. Compounding this knowledge gap, visa medical exams emphasize infectious diseases over psychological needs, resulting in almost absent pre-migration mental health data. Thus, while increasing U.S. immigration trends introduce significant mental health challenges, they remain insufficiently characterized in terms of prevalence, presentation, and trends.

To address these compounded gaps, a deeper understanding of these mental health characteristics is imperative for policymakers to design effective, culturally responsive strategies and to enhance the delivery of mental health care tailored to this population. Existing research focuses predominantly on post-arrival adaptation, neglecting the transitional “immigration applicant” phase, where legal and medical screening may exacerbate distress. It is precisely this overlooked phase that our study targets. By examining mental disorders among Chinese-American immigration applicants, this research provides urgently needed evidence to guide healthcare professionals and policymakers in developing targeted interventions.

## 2. Methods and materials

### 2.1. Study design and ethical considerations

This retrospective, cross-sectional study adhered to the principles of the Declaration of Helsinki and Good Clinical Practice guidelines. The ethical approval for this study was waived by the ethics committee of Shanghai International Travel Healthcare Center (SITHC). The reason for the waiver is that the study meets the criteria for ethical review exemption as stipulated by the relevant ethical guidelines and regulations. Due to the retrospective design, informed consent from participants was waived.

### 2.2. Data collection

The study evaluated health assessment records of Chinese-American immigrant applicants examined at the SITHC between January 2017 to December 2024. SITHC serves as the designated agency for U.S. immigrant health assessments in Eastern China, where mental health screening is a mandatory component of the evaluation process. The analysis included cases of individuals diagnosed with mental disorders during these assessments. Diagnoses were established by certified Chinese panel physicians using the criteria outlined in the Diagnostic and Statistical Manual of Mental Disorders, Fifth Edition, for purposes related to immigration and investigation. Key variables assessed in the study included age, gender, temporal changes in detection rates, and the distribution of mental disorders.

### 2.3. Statistical analysis

Data were analyzed using SPSS (version 26.0, IBM Corp., Chicago) and Microsoft Excel (Microsoft Corp., Redmond). The normality of continuous variables was assessed with the Kolmogorov–Smirnov test. Continuous data are reported as means ± standard deviations, while categorical data are expressed as frequencies and percentages. For between-group comparisons, analysis of variance (ANOVA) was used for continuous variables, and categorical variables were analyzed using either the chi-square test or Fisher exact test, depending on applicability. The Mann–Whitney *U*-test was applied for continuous variables that were not normally distributed. The Cochran–Mantel–Haenszel test was employed to examine differences across the total population while adjusting for temporal factors. Temporal trends in detection rates were evaluated using the Cochran–Armitage trend test. Statistical significance was defined as a two-sided *P*-value <.05.

### 2.4. Descriptive findings

#### 2.4.1. Characteristics of the sample

Between 2017 and 2024, 34,558 Chinese–American immigrant applicants underwent health evaluations at the SITHC, and were included in this study. The mean age of the participants was 32.7 years, with a standard deviation of 11.2 years. Mental disorders were diagnosed in 54 individuals, resulting in a prevalence rate of 1.56 per 1000 applicants. Among the 14,762 male applicants, 26 were identified with mental disorders (prevalence: 1.76 per 1000), whereas 28 cases were recorded among the 19,796 female applicants (prevalence: 1.41 per 1000). After adjusting for temporal variables, statistical analysis revealed no significant difference in prevalence rates between genders (χ^2^ = 0.53, *P* = .463). Regarding marital status, 36 affected applicants (66.7%) were married, and 18 (33.3%) were unmarried.

#### 2.4.2. Analysis of age distribution

Analysis of the age distribution (Table 1) demonstrated significant variation in the number of mental disorder cases across different age groups among Chinese-American immigrant applicants (χ^2^ = 19.352, *P* = .002). Specifically, higher frequencies were observed in individuals aged 15 years and younger (n = 14), those aged 15 to 30 years (n = 19), and those aged 75 years and older (n = 6) compared to other age categories. The groups aged 15 years and younger and 15 to 30 years accounted for 61.2% of all diagnosed cases. Furthermore, the prevalence rate in these 2 age groups was 2.8 per 1000 applicants, significantly exceeding the rate of 0.9 per 1000 applicants observed in the remaining age groups (χ^2^ = 17.091, *P* < .0001).

**Table 1:** Age distribution of mental disorders.

Age (yr)	Number of applicants (n)	The number of patients (n)	Detection rate (‰)
≤15	5030	14	2.78
15–30	6761	19	2.81
30–45	8162	8	0.98
45–60	6097	5	0.82
60–75	4561	2	0.44
>75	3947	6	1.52
Total	34,558	54	1.56

#### 2.4.3. Distribution of mental disorders

**Figure 1.**
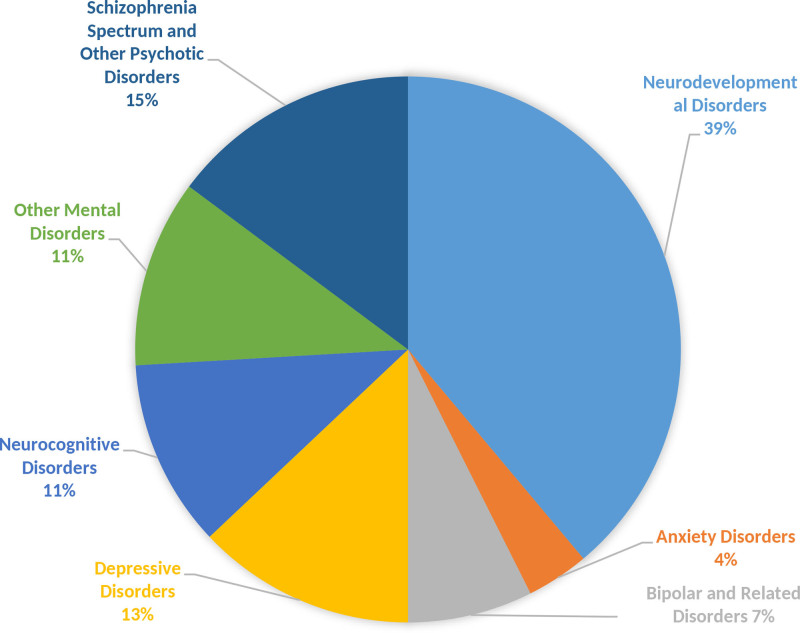
Distribution of mental disorders 2017–2024. This figure illustrates the distribution of mental disorders and their proportional (%) from 2017 to 2024. Column/row categories: Neurodevelopmental disorders, anxiety disorders, bipolar and related disorders, depressive disorders, neurocognitive disorders, other mental disorders, schizophrenia spectrum and other psychotic disorders. Data interpretation: Neurodevelopmental disorders show a 21.0% self-association. Associations with anxiety disorders: 2.0%, bipolar and related disorders: 4.0%, and so on. Data collection period: January 2017 to December 2024.

The results showed that among the 54 applicants diagnosed with mental disorders, neurodevelopmental disorders accounted for the largest share at 39%. This was followed by schizophrenia spectrum and other psychotic disorders at 15%, depressive disorders at 13%, and neurocognitive disorders at 11%. The proportions of bipolar and related disorders were relatively low at 7%, while anxiety disorders comprised 4%. Additionally, other mental disorders made up 7% of the total cases, as depicted in Figure 1.

#### 2.4.4. Evaluation of the level of disease

This study investigated the societal impact of mental disorders among immigrants, focusing on the severity of these conditions rather than their specific diagnostic classifications. The severity of mental disorders was determined based on the presence of harmful behavior, as outlined by the U.S. Centers for Disease Control and Prevention. According to U.S. Centers for Disease Control and Prevention guidelines, applicants may be rendered inadmissible if they exhibit a mental or physical disorder associated with harmful behavior. Harmful behavior encompasses actions stemming from a mental or physical disorder that result in significant psychological or physical harm to the individual or others, present a substantial threat to public health or safety, or cause extensive property damage.

Applicants diagnosed with a current mental or physical disorder, following the criteria established in the Diagnostic and Statistical Manual of Mental Disorders (DSM), and who exhibit harmful behavior are classified as Class A. In contrast, those diagnosed with such disorders but who do not display harmful behavior are designated as Class B. Among the cases assessed in this study, only 2 applicants (3.7%) were classified as Class A, with the remaining individuals categorized as Class B.

#### 2.4.5. The trend of detection rate

No statistically significant difference was found in the overall trend of the detection rate from 2017 to 2024 (Cochran–Armitage *Z* = −0.4004, *P* = .6889). However, an upward trend was observed between 2017 and 2021, followed by a decline from 2021 to 2024. The peak detection rate was recorded in 2021 at 3.95‰. This may be due to the relatively small sample size, as though the graphical representation shows a changing trend, statistical analysis did not reach significance.

For males, the detection rate similarly showed no significant increase (Cochran–Armitage *Z* = −2.642, *P* = .7917), with a rising trend noted from 2017 to 2021, after which the rate plateaued from 2014 onward.

**Figure 2.**
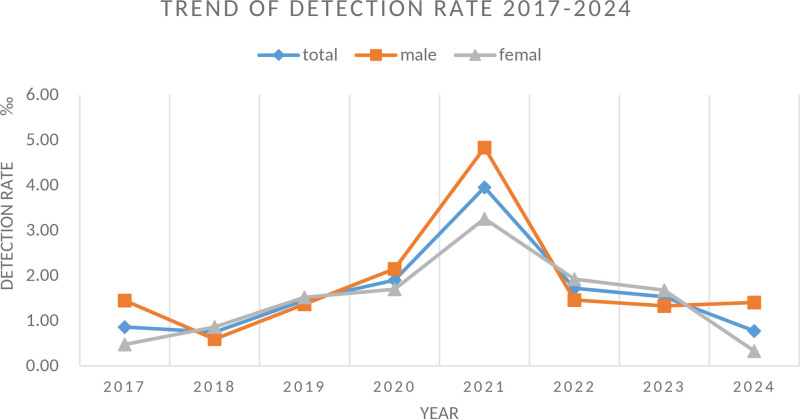
Trend of detection rate 2017–2024. This figure presents the annual detection rates of mental disorders from 2017 to 2024, categorized by total population, male, and female groups. Columns: Years (2017–2024). Rows: detection rate for all genders (total), male individuals (male), female individuals (female). Data Interpretation: Total detection rate peaked in 2021 (3.9‰) and showed fluctuations across the period. Male detection rates exhibited high variability, with the highest value in 2021 (4.8‰). Female detection rates showed a relative increase from 2017 to 2019, peaking in 2021 (3.2‰). Data collection period: January 2017 to December 2024.

The female detection rate exhibited a comparable pattern, with no significant increase overall (Cochran–Armitage *Z* = −2.922, *P* = .770). A growth trend was evident from 2017 to 2021, followed by a decline in subsequent years. The highest detection rate for females occurred in 2021, reaching 3.26‰. These trends are depicted in Figure 2.

## 3. Discussion

This retrospective survey offers valuable information regarding the mental health condition of Chinese–American immigrant applicants who underwent screening at the SITHC. The results indicated a very low detection rate of mental health disorders among them, with an incidence of merely 15.63 per 10,000 people from 2017 to 2024.

The elevated mental health risks observed among young unmarried applicants in our cohort (χ^2^ = 19.352, *P* = .002) align with vulnerability patterns identified in diverse migrant populations. Kobayashi T et al showed that the incidence of severe psychological distress (K6 ≥ 13 points) among female immigrants aged 20 to 44 is 3 times the average level in Japan, and unemployment and social isolation further exacerbate the risk (RR = 1.75).^[10]^ The high-risk group ≤ 30 years old identified in this study echoes this across cultures, confirming the decisive impact of age and migration stages (such as the period of occupational instability and family separation) on mental health. The phenomenon that only 2% of Chinese immigrants actively seek psychological services due to cultural stigma provides a key explanation for the extremely low detection rate (1.56‰) in this study,^[11]^ which is consistent with the research conclusions of Chen AW et al.^[12]^ It is important to note that the reported detection rate might not be an accurate representation of the prevalence of mental disorders. Immigrant groups, particularly those from Asian countries such as China, often face significant cultural stigma relating to mental illness. The stigma of mental health can result in the hiding of mental health problems since individuals and families might minimize or deny symptoms to evade social consequences or safeguard immigration status. This was witnessed during medical assessments, where panel physicians reported cases of undisclosed information. This, thus, poses a significant challenge for clinicians who are trying to diagnose and treat mental illness in immigrant populations effectively.

The incidence was far below the previously reported incidence rates of mental disorders in both developing nations and China. Research has shown that the rates of mental disorders in children and adolescents in developing countries range from 5 to 18%.^[13,14]^ For comparison, data obtained from Chinese surveys reveal a current rate of 9.3% and a lifetime rate of 16.6% in adults.^[15]^ The distribution of diagnoses in this study showed neurodevelopmental disorders to be the most common (39%), followed by schizophrenia spectrum and other psychotic disorders (15%), depressive disorders (13%), and neurocognitive disorders (11%). Bipolar disorders (7%), anxiety disorders (4%), and other mental disorders (7%) were less common, and there is a need to examine these patterns in greater detail.

The relatively lower prevalence observed in the present study is explainable in terms of the socio-economic characteristics of the immigrant population. Most of the applicants belonged to investment immigrants (53.2%) or marriage immigrants (9.4%), categories generally marked by superior levels of education, financial stability, and superior availability of healthcare.^[10]^ All these conditions are most likely to be associated with the decreased incidence of mental disorders in this group. Furthermore, variations in personal traits, diagnostic criteria, evaluation instruments, and research methods can explain the noted discrepancies in identification rates. So, the second source of the difference may lie in the screening scenarios: This study relies on the medical assessment framework of immigration physical examinations, which is more likely to identify “hard indicators” such as development history/psychotic symptoms; while community surveys (such as National Latino and Asian American Studies) rely on self-rating scales and are more sensitive to internalizing disorders (anxiety/depression).^[16]^

Improving the diagnostic ability of healthcare providers and making screening activities more culturally sensitive are essential for effectively tackling this issue. A temporal trend analysis of detection rates throughout the study period showed no significant overall trend; however, an increase was observed from 2017 to 2021, which decreased from 2021 to 2024. The spike in the year 2021 can be attributed to the launch of the Diagnostic and Statistical Manual of Mental Disorders, Fifth Edition, which led to increased awareness and visibility of the diagnosis and treatment of mental disorders in China during that time. Although these findings are promising, they highlight the necessity of thorough mental health treatment and training among immigrants, specifically following their initial arrival in the U.S. The inclination to mask mental illness can encroach on access to services in that Chinese immigrants and their families may shy away from admitting or seeking attention for their mental health concerns.

This reluctance may be a hindrance to diagnosis and treatment and highlights the necessity for culturally responsive healthcare systems that address the particular needs of immigrant communities. Upon arrival in the U.S., immigrants also have to contend with several challenges, including cultural adjustment, linguistic barriers, and socio-economic stressors, which can all exacerbate underlying mental illness. First-generation immigrants are twice as likely as native-born citizens to develop psychotic disorders, and social stressors related to migration – rather than genetic predisposition – are major contributing factors.^[17,18]^ Culture shock, language proficiency, and socio-economic conditions are key issues that will impact the immigrant’s mental state and should guide preventive interventions and therapeutic approaches.^[19]^ The identification of risk factors in mental disorders is a crucial answer to understanding the mental health needs of immigrant communities. This body of literature identifies risk factors that operate across different dimensions: individual (e.g., migration age, socio-economic position, and language competency), interpersonal (e.g., social support through families and adaptation to the new culture), and systemic (economic barriers to health care accessibility or the risk of discrimination).^[6,20,21]^

A younger age at the time of the study and being unmarried were found to have a positive association with having mental disorders, which thus aligns with generally available international data.^[18,22–26]^ Such results could further inform the establishment of targeted health services and policies tailored specifically toward satisfying the distinct needs of Chinese immigrants. The prevention of mental disorders is particularly well-placed to be effective where it targets groups identified as being at risk. Among these, neurodevelopmental disorders presented with the highest number of cases, with 39% prevalence, followed by schizophrenia and other psychotic disorders with 15%, depressive disorders with 13%, and neurocognitive disorders with 11%. This is in stark contrast to bipolar and anxiety disorders, which indicated relatively lower rates of prevalence. The findings represent implications for public health administrators in tailoring mental health services that will mainly address the unique needs of Chinese immigrant communities.

### 3.1. Limitations and future research

The study showed some limitations, besides using only one site as the focus area of study (East China), which renders it unrepresentative of Chinese–American immigrants in other parts of the geography. The variability in the regional distribution of healthcare access, socio-economic standing, and cultural attitudes toward mental health might limit how generalizable such findings might be.

In addition, the relatively low proportion of people found to have other mental disorders, notwithstanding the rigorous diagnostic procedure put in place, suggests that there is likely some underreporting. This could be due to the cultural stigma attached to mental illness or hesitation in treatment-seeking behavior – potentials that are typically present in immigrant populations.

The actual incidence of mental health disorders among this population may be even greater than what is recorded. Future studies in this field should look into a broader range of socio-political and economic dynamics at work in the emotional lives of immigrant populations. Important areas for further investigation include discrimination, social support networks, and healthcare services. It is also recommended that longitudinal studies of immigrant mental health trajectories be conducted in the U.S. to examine the effectiveness of targeted prevention and intervention programs.

Such initiatives would greatly inform our knowledge of this group’s mental disorders and allow plans to be drawn for evidence-based, culturally acceptable interventions.

## 4. Conclusion

The results of this study indicate a need for culturally focused mental health prevention interventions for young (under 30) and elderly (over 75) Chinese–American immigrants, 2 particularly vulnerable populations. These comparisons underscore that mental health profiles in immigration applicants cannot be extrapolated from general migrant studies. Future research must adopt culturally-adapted transdiagnostic tools to decode the complex vulnerability landscape in this phase of migration.

## Acknowledgments

The authors appreciate the generosity of the cohort participants who gave their effort and time to participate in this study. Meanwhile, the author would like to thank reviewers for their assistance in manuscript correction.

## Author contributions

**Conceptualization:** Zhiying Ju, Xuan Zhou, Peiqin Zhu, Peng Li, Jia Qin.

**Data curation:** Zhiying Ju, Jia Qin.

**Formal analysis:** Zhiying Ju, Peiqin Zhu, Weixin Wang, Jia Qin.

**Funding acquisition:** Zhiying Ju, Weixin Wang, Jia Qin.

**Investigation:** Zhiying Ju, Xuan Zhou, Peiqin Zhu, Peng Li, Jia Qin.

**Methodology:** Peiqin Zhu, Peng Li, Weixin Wang, Jia Qin.

**Project administration:** Xuan Zhou, Peiqin Zhu, Peng Li, Weixin Wang, Jia Qin.

**Resources:** Zhiying Ju, Peiqin Zhu, Weixin Wang, Jia Qin.

**Software:** Zhiying Ju, Xuan Zhou, Peiqin Zhu, Weixin Wang, Jia Qin.

**Supervision:** Jia Qin.

**Validation:** Zhiying Ju, Xuan Zhou, Peng Li, Weixin Wang, Jia Qin.

**Visualization:** Zhiying Ju.

**Writing – original draft:** Zhiying Ju, Xuan Zhou, Peiqin Zhu, Peng Li, Weixin Wang.

**Writing – review & editing:** Peiqin Zhu, Weixin Wang, Jia Qin.
